# Kinetics of RPR Decline in Pregnant Persons Treated for Syphilis in Pregnancy and Their Infants

**DOI:** 10.3390/pathogens13111010

**Published:** 2024-11-17

**Authors:** Danielle Schwartz, Alena Tse-Chang, Joan Robinson, Jennifer Gratrix, Petra Smyczek, Michael T. Hawkes

**Affiliations:** 1 Department of Medicine, University of Alberta, Edmonton, AB T6G 2R3, Canada; dsschwar@ualberta.ca; 2 Department of Pediatrics, University of Alberta, Edmonton, AB T6G 2R3, Canada; awtse@ualberta.ca (A.T.-C.);; 3 Sexually Transmitted Infections Services, Provincial, Population, and Public Health, Alberta Health Services, Edmonton, AB T5J 3E4, Canada; jennifer.gratrix@albertahealthservices.ca (J.G.);; 4 Department of Pediatrics, University of British Columbia, A5-179, 950 West 28th Ave., Vancouver, BC V5Z 4H4, Canada

**Keywords:** congenital syphilis, rapid plasma reagin, neonatal Fc receptor, antibody, kinetics

## Abstract

Congenital syphilis is a re-emerging infectious threat in areas of North America. The purpose of this study was to quantitatively describe the rate of decline of nontreponemal (rapid plasma reagin, RPR) titers in pregnant persons with syphilis and their infants. In a retrospective review, we included 120 pregnant persons with 563 reactive RPR measurements (median 5, range 2 to 11 per person) and 35 infants with 81 RPR measurements (median 2, range 2 to 6 per infant). First-order decay, second-order decay, and a mathematical model representing functional FcRn-mediated antibody recycling were fitted to individual patient RPR trajectories. The RPR titers decreased with a median half-life of 39 days (IQR 28–59) and 27 days (IQR 17–41) in birthing parents and infants, respectively. The half-life varied with the initial RPR titer, suggesting that the kinetics of RPR decline was not first-order. A mathematical model accounting for saturable antibody recycling explained the longevity of RPR reactivity, predicted the observed non-linear kinetics, and fit the empiric data well. In summary, RPR titers decline with a half-life of roughly one month; however, the elimination does not follow first-order kinetics. Saturable antibody recycling may explain the prolonged and non-linear elimination of nontreponemal antibodies.

## 1. Introduction

During the last decade, there has been a resurgence of infectious syphilis in numerous countries, including the USA and Canada [1]. This has been mirrored by a dramatic increase in congenital syphilis [1]. In the USA, the incidence of congenital syphilis infections increased every year since 2013, with 2268 congenital syphilis cases in 2021, the highest count since 1994 [2]. In Canada in 2022, 117 cases of confirmed early congenital syphilis were reported, representing a staggering 599% increase since 2018 [3].

Nontreponemal (lipoidal antigen) titers play a central role in monitoring the response to therapy in pregnancy and the management of the infant exposed to syphilis in utero. The rapid plasma reagin (RPR) is one of the primary nontreponemal (lipoidal antigen) tests used in the USA and Canada. The assay is a flocculation test that uses charcoal to aid in the detection of the antibody–antigen complexes that precipitate out of solution upon the addition of the lipoidal antigen [4]. Manual or automated detection procedures have been developed [5]. Endpoint dilution titers can be used to quantify the RPR, and represent a surrogate of the antibody concentration. Lipoidal antigens are a combination of cardiolipin, cholesterol, and phosphatidylcholine, constituent macromolecules of the host tissues as well as the *Treponema pallidum* cytoplasmic membrane [6]. Antibodies to the lipoidal antigens vary with disease activity, allowing them to be used as a test of cure. In pregnant persons diagnosed with syphilis and treated, a fourfold decrease in RPR titer provides reassurance of adequate treatment and influences management decisions for the infant [7]. In infants exposed to syphilis in utero but uninfected, reactive RPR titers, due to transplacentally acquired IgG immunoglobulin, may be followed after birth to ensure that they decline as expected as maternal antibodies are gradually cleared from the circulation [7]. In infants with confirmed congenital syphilis, guidelines recommend following RPR titers after treatment as a test of cure [7]. Despite the central role of nontreponemal titers in guiding treatment decisions and follow-up, few empiric data are available on the rate of RPR decline following treatment in pregnancy and in exposed uninfected or infected infants.

Antibodies that react to the lipoidal antigens may follow elimination kinetics similar to other native or synthetic antibodies. In general, antibodies may be eliminated via excretion or catabolism. Because of their large molecular size, little intact immunoglobulin is filtered by the kidney and excreted in the urine [8]. Instead, antibodies are predominantly eliminated via proteolysis after transit and sorting through endosomes [9]. Antibodies are internalized via fluid-mediated endocytosis where they bind to the Fc receptor of the neonate (FcRn) [9]. FcRn-unbound antibodies in the endosomes are trafficked to lysosomes, where they are degraded. FcRn-mediated cellular recycling rescues IgG from intracellular degradation, prolonging the serum half-life. Thus, the serum half-life of IgG (23 days) is substantially longer than other immunoglobulin isotypes (2.5–6 days) [8]. Because of their complex handling, the elimination of antibodies against lipoidal antigens may also follow non-linear pharmacokinetics [10,11,12,13].

The objective of this study was to quantitatively describe the rate of decline of nontreponemal titers in treated pregnant persons and their infants. Understanding the expected kinetics of RPR titer over time may inform clinical practice through early detection of treatment failure or re-infection, which may prompt re-treatment.

## 2. Methods

### 2.1. Study Design

This was a retrospective cohort study. Clinical data (1 January 2015 through 31 December 2021) were abstracted from a public health database maintained by the Sexually Transmitted Infections Services, Provincial, Population, and Public Health, Alberta Health Services (STI Services). We began by selecting unique parent–infant pairs if infectious syphilis was diagnosed during the pregnancy. Individual measurements of the RPR titer from the pregnant parent were included in the analysis only if the measurement was within the 9-month interval prior to delivery to the first month post-partum. Non-reactive tests were not included as they were not considered quantifiable. From the cohort of eligible parent–infant pairs, individual parents or infants were included only if they had a fourfold (or greater) documented decrease in RPR titer. Individual parents or infants were excluded if there was a fourfold (or greater) rise in RPR, suggestive of treatment failure or re-infection. Congenital syphilis among study infants was classified according to the CDC guidelines [7]. as: (1) confirmed proven or highly probable; (2) possible; or (3) less likely.

### 2.2. Statistical Analysis

We assumed that the inverse of the RPR titer was proportional to the antibody concentration. The data were summarized using the number (n) and percentage for binary variables and median with interquartile range (IQR) for continuous variables.

### 2.3. Non-Linear Mixed-Effects Model

First- and second-order decay kinetics were fitted to the observed data using non-linear mixed-effects regression (package *nlme* [14,15]. in the R statistical environment). The error term was assumed to be normally distributed on the base-2 logarithmic (${log}_{2}$) scale. This assumption is commensurate with biological and technical aspects of the RPR endpoint dilution assay, in which serial twofold dilutions are performed on the patient serum until the sample is non-reactive. A twofold difference in RPR titer is within the error of the assay. Thus, the equations for first- and second-order decay were converted to a ${log}_{2}$ scale, as follows:

#### 2.3.1. First-Order Kinetics

$$\frac{dc}{dt}=-k_{1}\cdot c$$

$$c={c_{0}\cdot e}^{-k_{1}\cdot t}$$

$${log}_{2}\left(c\right)={log}_{2}\left(c_{0}\right)-k_{1}\cdot t$$

$${log}_{2}\left(c_{i,j}\right)={log}_{2}\left(c_{0,i}\right)-k_{1,i}\cdot t_{i,j}+\varepsilon_{i,j}$$

where:

$c_{i,j}$ is the concentration $\left(\frac{1}{titer}\right)$ with longitudinal measurements over time for patient i
i is the patient index
j is the index for the $j^{th}$ measurement on patient i
$c_{0,i}$ is the initial concentration for patient i
$k_{1,i}$ is the first-order rate constant for patient i, assumed to be normally distributed, $k_{1,i}\sim N(k_{1},\sigma_{k_{1}}^{2})$
$t_{i,j}$ is the time elapsed since the first RPR measurement
$\varepsilon_{i,j}$ is the residual, assumed to be normally distributed, $\varepsilon_{i,j}\sim N(0,\sigma^{2})$

#### 2.3.2. Second-Order Kinetics

$$\frac{dc}{dt}=-k_{2}\cdot c^{2}$$

$$\frac{1}{c}=\frac{1}{c_{0}}+k_{2}\cdot t$$

$$c=\frac{c_{0}}{1+c_{0}\cdot k_{2}\cdot t}$$

$${log}_{2}\left(c\right)={log}_{2}\left(c_{0}\right)-{log}_{2}(1+c_{0}\cdot k_{2}\cdot t)$$

$${log}_{2}\left(c_{i,j}\right)={log}_{2}\left(c_{0,i}\right)-{log}_{2}(1+c_{0,i}\cdot k_{2,i}\cdot t_{i,j})+\varepsilon_{i,j}$$

where:

$c_{i,j}$ is the concentration $\left(\frac{1}{titer}\right)$ with longitudinal measurements over time for patient i
i is the patient index
j is the index for the $j^{th}$ measurement on patient i
$c_{0,i}$ is the initial RPR titer for patient i
$k_{2,i}$ is the first-order rate constant for patient i, assumed to be normally distributed, $k_{2,i}\sim N(k_{2},\sigma_{k_{2}}^{2})$
$t_{i,j}$ is the time elapsed since the first RPR measurement
$\varepsilon_{i,j}$ is the residual, assumed to be normally distributed, $\varepsilon_{i,j}\sim N(0,\sigma^{2})$

Maximum likelihood estimation was used to find the optimum values of k, $\sigma_{k}$, and σ for each of the first- and second-order models. Data from parents and infants were fitted separately. The model quality was assessed using the Aikake information criterion (AIC). Of note, the first- and second-order models both used a single parameter (k) to describe the longitudinal trajectory.

Model assumptions were checked by visual inspection of residual plots.

### 2.4. Antibody Recycling Model

#### 2.4.1. Biological Rationale

The catabolism and recycling of IgG occurs adjacent to the vascular space, most likely in the endothelium and parenchymal cells of organs with fenestrated endothelia [10]. Therefore, we followed the simplification from a previous study [10], pooling these sites into a single compartment, the vascular space ($V_{1}$), assuming that the measurable plasma level rapidly equilibrated with the cellular and subcellular concentrations. A kinetic model was used, representing the situation within the endosome-rich endothelial cell, into which plasma IgG is pinocytosed. FcRn binding takes place after acidic sorting endosomes are formed. Functional catabolism (cat) and receptor-mediated recycling (rmr) of IgG were modeled by the parameters $k_{cat}$ and $k_{rmr}$, respectively. FcRn-bound IgG is recycled to the vascular space whereas unbound IgG is degraded. The fractional rate of FcRn-mediated recycling was assumed to be saturable, following classical Michaelis–Menten kinetics [10].

#### 2.4.2. Mathematical Model

The mass balance (per kg of body mass) on the RPR antibody can be expressed with the following differential equation:

$$\frac{d(C\cdot V_{1})}{dt}=J_{pro}-k_{cat}\cdot (C\cdot V_{1})$$

where:

C is the concentration of RPR antibodies
$V_{1}$ is the volume of the vascular space (mL/kg) [10].
$J_{pro}$ is the rate of production of antibodies (mg/d/kg) [10].
$k_{cat}$ is the fractional catabolic rate (d^−1^) [10].

Assuming that a proportion of the antibodies will be recycled by FcRn, the following equation follows: [10].

$$k_{cat}=k_{int}-k_{rmr}$$

where:

$k_{int}$ is the fractional intrinsic catabolic rate (d^−1^)
$k_{rmr}$ is the fractional receptor-mediated recycling rate (d^−1^).

The FcRn-mediated recycling is assumed to follow saturable (Michaelis–Menten) kinetics: [10].

$$\frac{dC}{dt}=\frac{J_{max}\cdot C}{\frac{K_{m}}{\theta }+C}$$

where:

$J_{max}$ is the maximal rate of FcRn-mediated recycling (mg/d/kg)
$K_{m}$ is the Michaelis–Menten constant (mg/mL)
θ is a conversion factor for antibody concentration to RPR titer (mg/mL/titer unit).

This yields the following fractional recycling rate [10]:

$$k_{rmr}=\frac{J_{max}}{V_{1}\cdot (\frac{K_{m}}{\theta }+C)}$$

And net catabolic rate:

$$k_{cat}=k_{int}-\frac{J_{max}}{V_{1}\cdot (\frac{K_{m}}{\theta }+C)}$$

Assuming that the vascular volume is constant,

$$\frac{dC}{dt}=\frac{J_{pro}}{V_{1}}-k_{int}\cdot C+\frac{J_{max}\cdot C}{V_{1}\cdot (\frac{K_{m}}{\theta }+C)}$$

The system was assumed to be at a steady state at the time of the initial RPR measurement. At this point, the influx of Ab ($J_{pro}$) into the serum compartment was due to production by maternal or infant B-lymphocytes, or the transfer of maternal IgG across the placenta. After treatment or birth, we assumed a stepwise cessation of antibody production or transfer. This led to the following differential equation for the change in concentration:

$$\frac{dC}{dt}=-k_{int}\cdot C+\frac{J_{max}\cdot C}{V_{1}\cdot (\frac{K_{m}}{\theta }+C)}$$

This is a separable differential equation with an analytical solution; however, the solution was cumbersome and transcendental. Therefore, we used numerical methods (package *deSolve* [16] in the R statistical environment, version 4.4.1) to solve the ordinary differential equation (initial value problem).

The values of the parameters were taken from past publications, with the exception of θ, representing a conversion factor from RPR titer to antibody concentration. This value was obtained by calibrating the model to the observed data. The values of $J_{max}$ and $K_{m}$ from a previous study [10] yielded a good fit to the observed data. These values were held constant for subsequent analyses. The value of $k_{int}$ was allowed to vary between patients, in order to provide the best fit to the individual patient trajectories. The value of $k_{int}$ was chosen for each patient that minimized the sum of squared residuals (ordinary least squares regression).

To compare the goodness-of-fit of the antibody recycling model to that of the first- and second-order decay models, we used the sum of squared errors (SSE), where the error (residuals) was the difference between the observed titer and the predicted titer at the same time, based on the initial concentration. For comparability, the SSE was re-calculated for the first- and second-order models fitted to the data using ordinary least squares regression (as was used for the antibody recycling model). Of note, the number of parameters in each model was one, such that the SSE could be compared directly in order to assess the model fit.

### 2.5. Ethics Approval

The study protocol was approved by the University of Alberta Health Research Ethics Board (Pro00117243). Parental consent was waived as this was a retrospective chart review.

## 3. Results

Of 291 parent–infant pairs with syphilis detected during the pregnancy, we included 121 pregnant persons with 569 reactive RPR measurements (median 5, range 2 to 11 per person). The remaining pregnant persons did not have a documented fourfold decrease in RPR or had a fourfold rise in RPR. In addition, we included 35 infants with 81 RPR measurements (median 2, range 2 to 6 per infant). The remaining infants were not included as they did not have a documented fourfold decrease in RPR titer. The patient characteristics are shown in Table 1.

### 3.1. Serum Half-Life of RPR Titers

**Figure 1:**
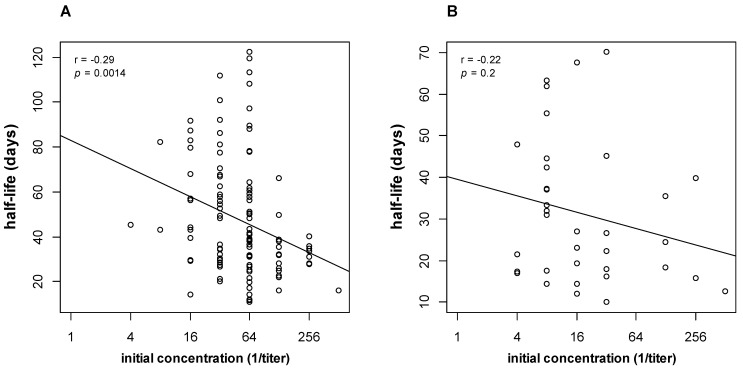
RPR titer half-life depends on the initial RPR. (**A**) Parental data, showing a shorter half-life when the initial titer is elevated. (**B**) Infant data, with a similar trend, although the correlation did not reach statistical significance. The Pearson correlation coefficient (r) with its *p*-value is shown for each plot. Each circle represents the half-life for an individual patient. The solid line represents the best fit regression line.

Among pregnant parents, the RPR titers decreased with a half-life of 39 days (IQR 28–59). Among infants, the RPR half-life was median 27 days (IQR 17–41). Among matched parent–infant pairs, we did not detect a statistically significant difference in half-life (*p* = 0.65). We did not detect a statistically significant difference in half-life among infants classified as confirmed proven or highly probable, possible, or less likely to have congenital syphilis (*p* = 0.95). The half-life varied with the initial RPR titer (Figure 1), suggesting that the kinetics of RPR decline was not first-order and that more complex pharmacokinetic models were required to quantitatively describe the longitudinal RPR titers.

### 3.2. Mathematical Models of RPR Titer Decay

We next explored several pharmacokinetic models: (1) first-order decay; (2) second-order decay; and (3) saturable antibody recycling.

**Figure 2:**
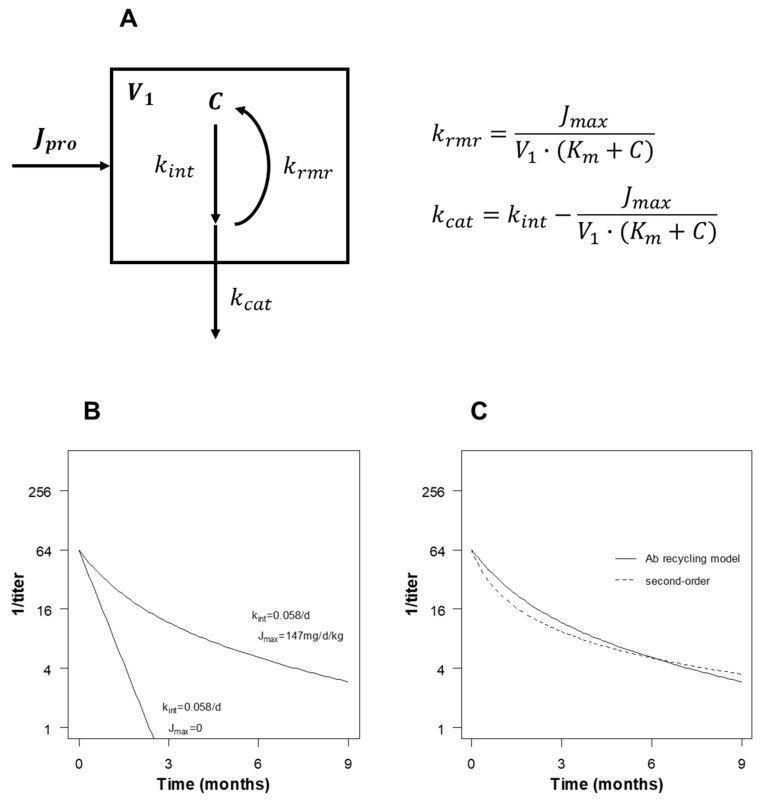
Antibody recycling model. (**A**) Schematic diagram for the mathematical model. Flux ($J_{pro}$) of nontreponemal antibody into the vascular space ($V_{1}$) due to production of antibody by B-cells or transplacental transfer of antibodies (for infants). Antibody is eliminated with fractional intrinsic catabolic rate $k_{int}$ and recycled at fractional receptor-mediated recycling rate $k_{rmr}$, yielding a net rate of catabolism represented by $k_{cat}.$ The rate of recycling was assumed to be saturable, following Michaelis–Menten enzyme kinetics, with formulae as displayed. (**B**) The model recapitulated the expected prolongation of antibody longevity with FcRn-mediated recycling. (**C**) The non-linear behavior of the model could be well approximated by second-order decay.

**Figure 3:**
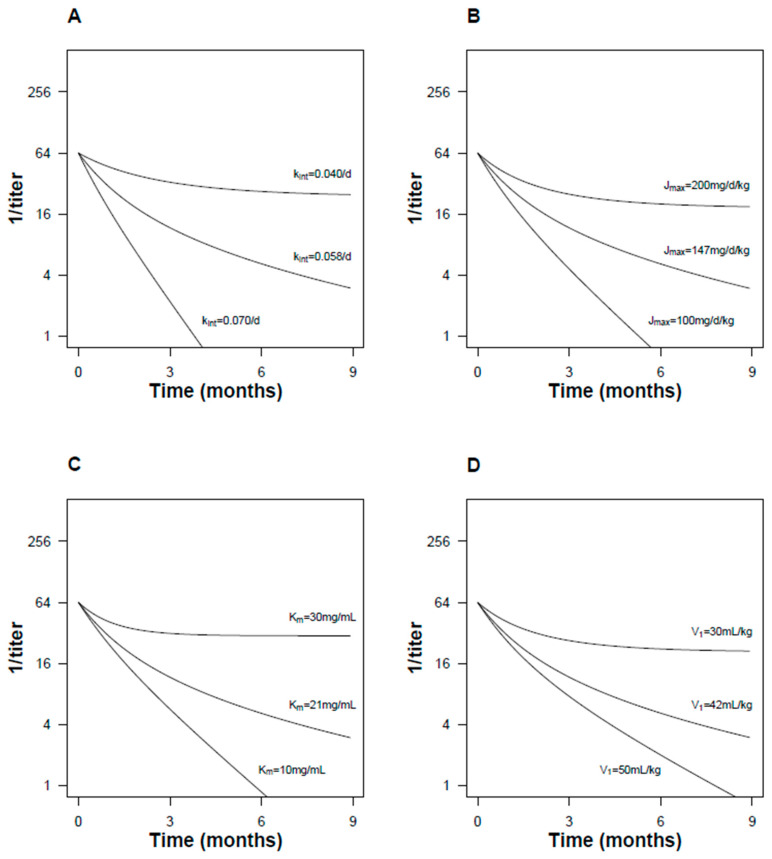
Antibody recycling model: effect of changing model parameters. (**A**) Increasing the fractional intrinsic catabolic rate ($k_{int}$) increased the rate of elimination. (**B**) Increasing the maximum recycling rate ($J_{max}$) reduced the elimination rate, and resulted in substantial deviation from linear (first-order) elimination kinetics. (**C**) Effect of changing the Michaelis–Menten constant ($K_{m}$). (**D**) Effect of changing the distribution volume ($V_{1}$).

**Figure 4:**
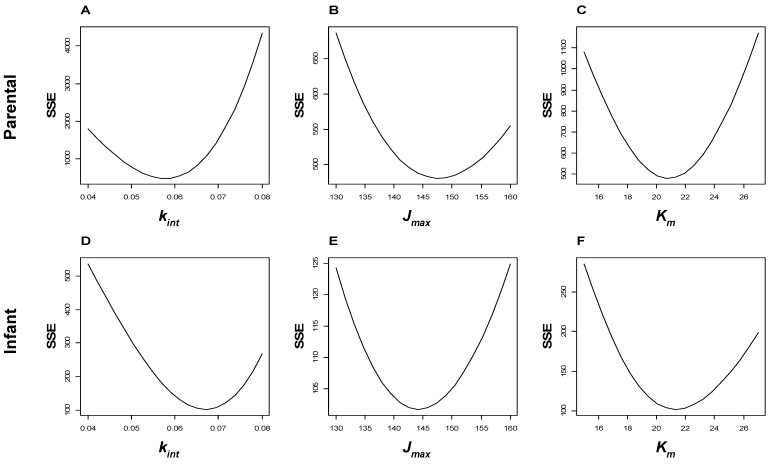
Antibody recycling model: parameter optimization. The model was calibrated to the maternal data (Panels (**A**–**C**)) and infant data (Panels (**D**–**F**)) separately. The parameters were systematically varied, the model predictions were compared to the observed data, and the sum of squared errors (SSE) was calculated. Parameter values were chosen to minimize the SSE. The local minima in the SSE are shown, providing a graphical display of the best-fit model parameters. For the intrinsic catabolic rate ($k_{int}$, Panels (**A**,**D**)), the optimal value differed between pregnant parents ($k_{int}$ = 0.060 d^−1^) and infants ($k_{int}$ = 0.074 d^−1^) and was substantially different from a published value of the intrinsic catabolic rate of IgG $(k_{int}$ = 0.18 d^−1^) [17]. On the other hand, the maximum recycling rate for the FcRn receptor ($J_{max}$, Panels (**B**,**E**)) was similar in pregnant parents and infants and similar to the published $J_{max}$ for IgG (147 mg/d/kg) [17]. For subsequent model fitting, this parameter was held constant at 147 mg/d/kg. Likewise, the Michaelis–Menten constant ($K_{m}$, Panels (**C**,**F**)) was similar in parents and infants and similar to the $K_{m}$ for IgG (21.0 mg/mL) [17]. This value was also held constant for subsequent model fitting.

The saturable antibody recycling model, based on a previous study [10], included terms for concentration-dependent Ab elimination and FcRn-mediated antibody recycling (Figure 2A). Qualitatively, the model explained declining RPR titers over time and the increase in antibody longevity by FcRn recycling (Figure 2B). The model predicted non-linear kinetics of antibody decay on a semi-logarithmic scale, which could be approximated by a simpler second-order decay model (Figure 2C). Varying the model parameters produced qualitatively intuitive changes in the elimination kinetics (Figure 3). The model was calibrated to the empiric data (Figure 4).

**Figure 5:**
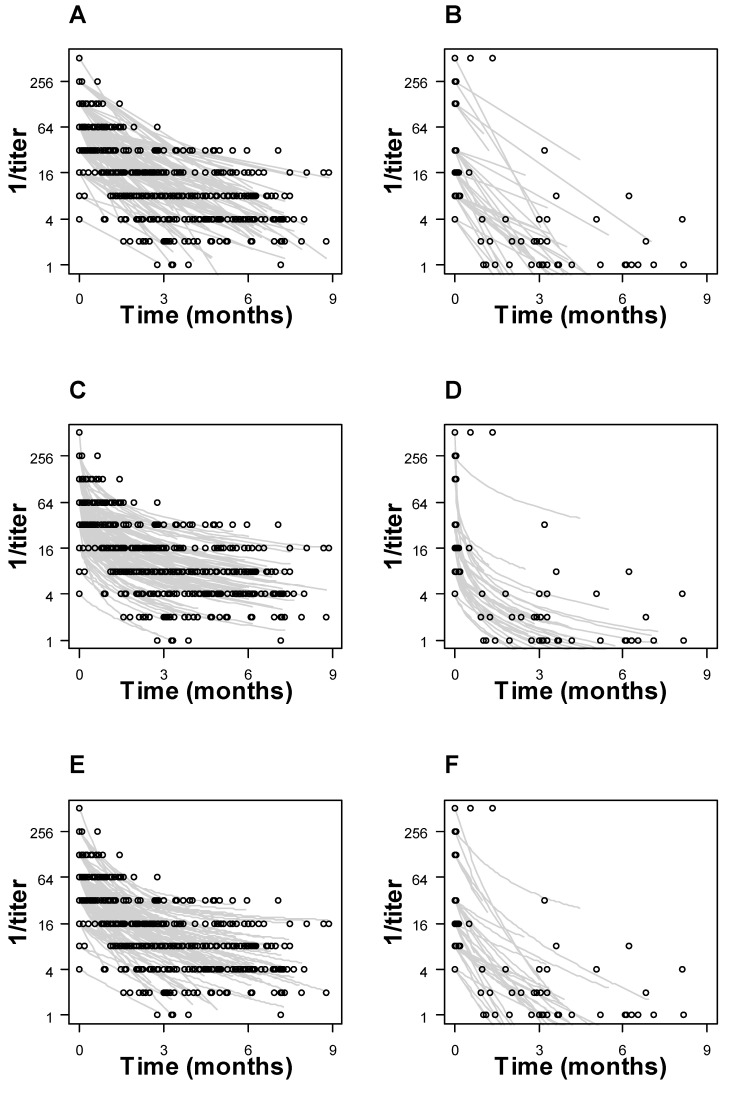
First-order, second-order, and antibody recycling models fitted to individual patient RPR trajectories. Maternal data (Panels (**A**,**C**,**E**)) and infant data (Panels (**B**,**D**,**F**)) were plotted separately. For each patient, the kinetic parameter ($k_{1}$ for first-order, $k_{2}$ for second-order, and $k_{int}$ for antibody recycling model) was chosen to minimize the SSE. The fitted curves are shown as gray lines and observed data as empty circles.

**Figure 6:**
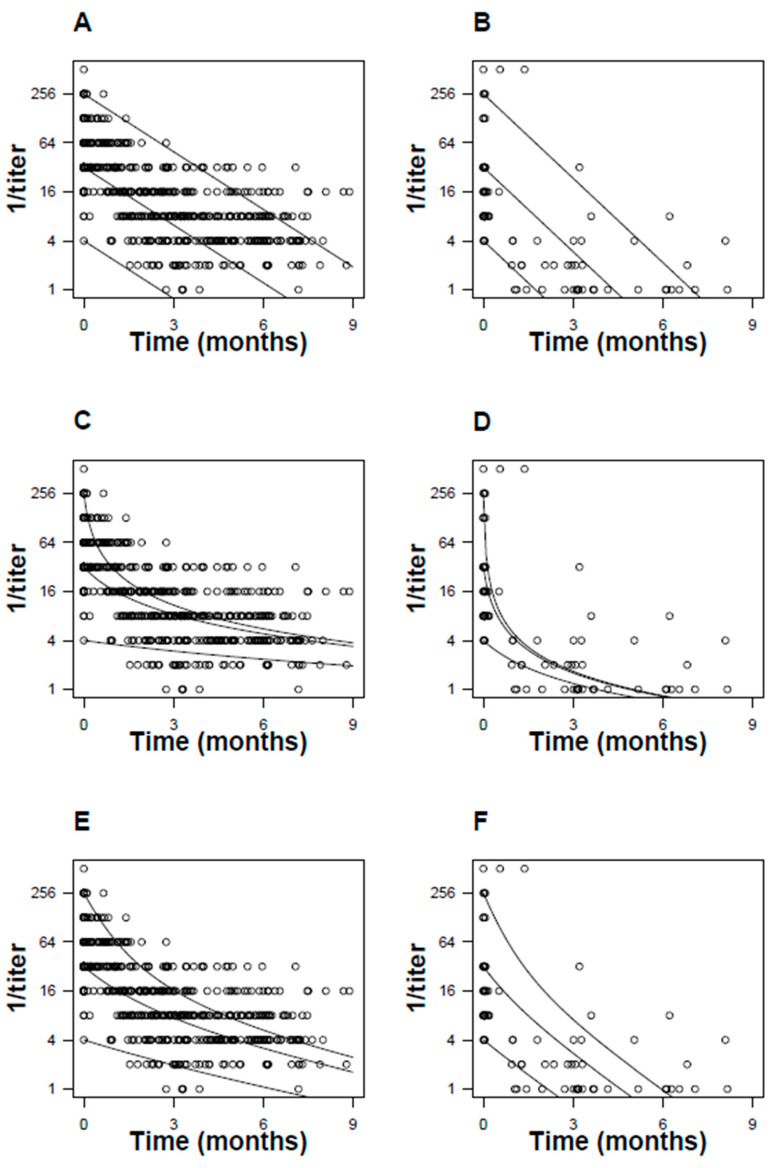
First-order, second-order, and antibody recycling model of RPR kinetics. Maternal data (Panels (**A**,**C**,**E**)) and infant data (Panels (**B**,**D**,**F**)) are plotted separately. After fitting the models to individual patient data, the median parameter for the cohort was used to construct best-fit curves at several initial RPR titers (solid lines). Circles represent individual measurements of RPR.

First-order, second-order, and antibody recycling models were fitted to the longitudinal RPR trajectory of individual patients (Figure 5). Summary curves for the cohort were generated separately for pregnant parents and infants (Figure 6). Model prediction error, estimated using the AIC, was 1300 vs. 1200 for first-order vs. second-order models (parental RPR titers) and 180 vs. 150 (infant RPR titers). The SSE was 220, 150, and 150 (parental RPR titers) and 16, 20, and 19 (infant RPR titers) for first-order, second-order, and antibody recycling models, respectively.

### 3.3. Serum Half-Life as a Function of Initial (Birth) RPR Titer

Because the half-life was not constant, we used the antibody recycling model to construct tables of the predicted half-life, time to fourfold reduction, and time to seroreversion for different initial RPR titers (Table 2).

## 4. Discussion

Here we describe the kinetics of decline of RPR titers in pregnant persons with treated syphilis and infants exposed to syphilis in utero. The serum half-life was 39 days (IQR 28–59) for pregnant parents and 27 days (IQR 17–41) for infants, but varied according to the initial RPR. Elimination did not obey first-order kinetics, but could be modeled with saturable FcRn-mediated antibody recycling. Our analysis is noteworthy for applying pharmacokinetic models to longitudinal RPR titers, which revealed substantial deviation from the usual assumptions about RPR titer decline.

The serum half-life varied with the initial RPR titer (Figure 1), which is not consistent with first-order elimination kinetics. Non-linear decay (in the semi-logarithmic plane) could be explained by saturable FcRn antibody recycling, as modeled by the antibody recycling model (Figure 2). Saturable antibody recycling was recognized as early as the 1960s, prior to the discovery and characterization of the FcRn receptor [18]. This behavior was sufficient to explain the shorter half-life at high initial RPR titer and was modeled with classical Michaelis–Menten kinetics, as in a previous study [10]. This non-linear behavior was well approximated by a simpler second-order decay model (Figure 2C). Indeed, second-order decay models provided a better fit to the empiric data than first-order models, based on the AIC. Antibody kinetics are frequently described by a serum half-life [19,20,21] time to fourfold decline [7], or time to seroreversion [22], without accounting for the initial RPR, which implicitly assumes first-order elimination kinetics (elimination rate independent of concentration). Our findings suggest that the elimination pathways of antibodies to lipoidal antigen may follow a more complex pattern. This observation was made possible because of the larger sample size relative to past studies, careful case selection, and detailed longitudinal analysis.

The long serum half-life was a noteworthy feature of the longitudinal RPR titers in our study. In clinical practice, the remarkable longevity of IgG, relative to other serum proteins, is well recognized. Transplacentally acquired maternal IgG to measles [20], rubella [23], varicella [23], and dengue virus [24] wane to levels below a protective threshold over a period of 6–12 months and may interfere with infant vaccination. Antibodies to HIV of maternal origin may be detectable in the infant serum for 18–24 months [22], with implications for diagnosis or vertical HIV transmission. By engineering the amino acid sequence of the FcRn binding region, the serum half-life of monoclonal antibodies can be substantially extended, as exemplified by the recently licensed RSV passive immunoprophylactic agent nirsevimab [25].

The recycling of RPR antibodies may also provide insight into the “serofast” state and “serologic non-responders” [26]. In the pre-antibiotic era, the term “serofast” was used to describe RPR titers that failed to become non-reactive. This had clinical implications since persons with early syphilis who remained serofast after treatment had a higher rate of neurologic complications [26]. In the modern antibiotic era, a fourfold decline in nontreponemal titers (rather than complete seroreversion), predicts a satisfactory clinical response. Patients who fail to achieve a fourfold reduction are called “serologic non-responders” [26]. Well-defined clinical conditions (e.g., HIV infection) are associated with serologic non-response due to polyclonal B-cell activation independent of *T. pallidum* disease activity [26]. Our study provides another possible explanation for delayed serologic response and prolonged serofast state after effective treatment, i.e., antibody recycling. At low concentrations, a high proportion of RPR antibodies are likely to be returned to the circulation via FcRn-mediated recycling, leading to non-linear decay and a long “tail” of antibody that may be detectable long after the eradication of viable spirochetes.

Our findings may have clinical implications for the early detection of treatment failure or re-infection. Greater accuracy in describing the trajectory of RPR titers after curative treatment or after delivery (in the case of exposed, uninfected infants) may help identify deviations from this expected course, which may signal a need for re-treatment. Using the antibody recycling model, we generated clinically relevant estimates of the half-life, time to fourfold reduction, and time to seroreversion from different initial RPR titers (Table 2). It may be observed that a fourfold reduction in RPR titer occurs within 190 days in 95% of children and pregnant women, for all RPR titers. Therefore, as a “real-world” rule of thumb, further investigation may be warranted when RPR titers fail to decrease fourfold within 6 months of therapy. This may have clinical utility as a benchmark against which follow-up RPR titers can be evaluated. On the other hand, the wide confidence intervals, owing to large patient-to-patient variability in longitudinal RPR trajectories, limited the precision of these estimates. Thus, the clinical implications of our findings for the interpretation of RPR titers as a test of cure warrant further study.

Our study has several limitations. The longitudinal data were sparse (median 5 per pregnant patient and 2 per infant). The sample size was small, particularly for the infant cohort (N = 35), which may limit the generalizability of the findings. The laboratory error of the assay was large and proportional to the RPR titers (±twofold dilution). Future studies with frequent sampling of the RPR longitudinally, larger patient numbers, and higher precision assays would be needed to validate our findings and define RPR kinetics more exactly. Model specification may be another source of error because we made several simplifying assumptions. More complex models of IgG pharmacokinetics have been published, which account for the distribution of antibodies into multiple compartments [13], pinocytosis rate [11], pH-dependent FcRn binding within the endosome [11], and organ-specific antibody handling [11]. Although such models could be used to more exhaustively reflect biological processes, the large number of parameters and uncertainty in their estimates may not be justified for modeling in vivo RPR data, with its inherent imprecision. Of note, our goal in applying a simplified pharmacokinetic model was to investigate the observed non-linear elimination kinetics and to determine whether this could be explained by FcRn-mediated antibody recycling, rather than to recapitulate biological processes.

In summary, our analysis of RPR decline in pregnancy and infancy provides insights into the complex elimination kinetics of antibody to nontreponemal (lipoidal) antigens. Future studies analyzing longitudinal RPR titers should consider non-linear kinetics. The application of these findings to clinical practice will need to balance the complexity of RPR elimination kinetics on the one hand with the real-world utility of simplified decision rules on the other. Given the rising incidence of syphilis in pregnancy, our study also draws attention to the need for more precise quantitative assays to monitor response to therapy.

## Figures and Tables

**Table 1:** Parental and infant characteristics.

Characteristic	
***Pregnant parent***	**(N = 121)**
Age [yr], median (IQR)	27 (23–31)
Parental treatment (initial course)	
Benzathine penicillin (1 dose)	18 (15)
Benzathine penicillin (2 doses)	92 (76)
Benzathine penicillin (3 doses)	11 (9.1)
Re-treatment	43 (36)
Mode of Delivery	
Vaginal	39 (32)
C-section (elective)	19 (16)
C-section (emergency)	7 (5.8)
Unknown	56 (46)
***Infant***	**(N = 35)**
Sex	
Female	14 (40)
Male	21 (60)
Gestational age [wk], median (IQR)	
Term (≥37 weeks)	27 (79)
Pre-term (<37 weeks)	7 (21)
Unknown	1 (2.9)
Congenital syphilis classification (%)	
Less likely	9 (26)
Possible	14 (40)
Confirmed proven or highly probable,	12 (34)
Infant treatment	
Penicillin G	28 (80)
Not treated	6 (17)
Unknown	1 (2.9)

Values represent n (%) unless otherwise stated.

**Table 2:** Antibody recycling model predictions for RPR half-life ($t_{1/2}$), time to fourfold reduction in initial titer ($t_{1/4}$), and time to seroreversion ($t_{revert}$) in pregnant parents and infants.

Initial RPR (1/Titer)		Parental			Infant	
	$t_{1/2}$	$t_{1/4}$	$t_{revert}$	$t_{1/2}$	$t_{1/4}$	$t_{revert}$
**512**	35 (2.2–150)	51 (3.4–180)	470 (120–1900)	11 (1.0–31)	22 (7.4–54)	200 (100–1500)
**256**	42 (1.8–180)	58 (4.2–180)	460 (120–1900)	13 (1.4–42)	25 (7.0–69)	190 (94–1600)
**128**	52 (2.2–180)	74 (4.4–190)	430 (110–1800)	14 (1.8–51)	29 (8.8–96)	180 (88–1600)
**64**	63 (3.8–190)	78 (6.0–190)	390 (84–1900)	17 (1.6–85)	36 (12–140)	160 (80–1600)
**32**	73 (4.2–190)	86 (5.2–190)	370 (58–1900)	22 (2.8–120)	43 (12–160)	150 (66–1500)
**16**	77 (4.4–190)	98 (8.6–190)	380 (44–1900)	23 (2.4–160)	47 (14–160)	140 (60–1600)
**8**	82 (3.6–190)	89 (6.6–190)	290 (26–1800)	25 (1.6–150)	52 (14–180)	110 (42–1500)
**4**	83 (4.0–190)	89 (5.0–190)	240 (16–1800)	27 (2.0–150)	52 (13–190)	86 (24–1300)

Values in the table represent time in days (95% confidence interval).

## Data Availability

Data will be provided upon reasonable request to the corresponding author.
